# Effect of Gamma Radiation on the Processability of New and Recycled PA-6 Polymers

**DOI:** 10.3390/polym15030613

**Published:** 2023-01-25

**Authors:** Carlos González Niño, Julio Vidal, Martina Del Cerro, Lucía Royo-Pascual, Gonzalo Murillo-Ciordia, Pere Castell

**Affiliations:** 1 Fundación CIRCE (Research Centre for Energy Resources and Consumption), Avenida Ranillas, Edificio Dinamiza 3D, 50018 Zaragoza, Spain; 2 Fundación Aitiip, Polígono Industrial Empresarium C/Romero Nº 12, 50720 Zaragoza, Spain

**Keywords:** polymer irradiation, gamma radiation, polymer recycling, circular economy, polyamides

## Abstract

The growing quantities of plastic waste have raised environmental concerns, with almost a quarter of disposed plastics being sent to landfill. This has motivated research efforts into various recycling technologies to ease dependence on fossil resources, increasing circularity. Irradiation of various kinds, such as electron beam, beta and gamma rays, has been studied in the past as a way of revamping end-of-life polymer properties. The present work focuses on the effects of gamma radiation on the processability of new and recycled polymers, which is intimately linked with their rheological properties. In this study, both virgin and recycled polymers were irradiated under different radiation doses and the effects of the radiation on their viscosity assessed and compared. Results were analyzed making use of different theoretical relationships, and the causes of the changes in rheology were investigated by means of various characterization techniques, such as GPC, FTIR, EPR and DSC. Finally, the rheological curves of all samples were fitted to the Ostwald–de Waele relationship and the dependence of its parameters on the absorbed dose fitted to a function.

## 1. Introduction

The increasing amount of postconsumer and postindustrial plastic waste poses a global challenge that needs to be properly addressed in order to increase circularity, reduce greenhouse gas emissions and ease dependence on fossil resources [1]. In fact, policies all around the globe exist in pursuit of this objective, especially within Europe where the European commission has stablished a strategy towards a 50% cut in plastic pollution [2]. More than 29 Mtons of postconsumer plastic waste were collected in the EU27+3 in 2020 alone, with over 23% being sent to landfill. However, the proportion of plastics being recycled in the EU27+3 is experiencing a steady growth with an increase of 69% from 2006 to 2020 [3]. A great variety of types of polymeric structures are found among the different plastic materials that are collected, sorted, washed and recycled. In some cases, the interest in recycling the materials lies in the amount of polymer that is used and the vast amounts that are produced yearly, as is the case for PE, PP and PET. In other cases, the interest in recycling materials arises from the value point of view rather than from the quantitative, which in comparison to the previously mentioned materials might seem negligible. Polyamide 6 is a semicrystalline polymer known for its good thermal and mechanical properties that is used in a wide range of applications and industries, such as automotive, aeronautical, electronic, textile and medical; this makes PA-6 a valuable material used in high-value sectors. Its capacities and excellent mechanical properties are the reason behind the increasing demand for PA-6 worldwide. The global yearly demand for polyamides is in the range of million tons, with production continuously growing [3,4], and thus, their recycling can have a big impact in terms of circularity. Chemical recycling is a common and growing approach for polyamides, where the polymer is transformed back into its monomer constituents before synthetizing new plastic again. In the case of PA-6, chemical recycling techniques include hydrolysis [5], organocatalyzed depolymerization in ionic liquids [6], aminolysis [7] and alcoholysis/glycolysis [8], among others. Still, these rely on the use of different substances in combination with the polymer such as an initiator or fillers, which might result in the release of toxic compounds during reaction, with some authors having discarded it as a viable alternative for plastic recycling [9]. Nevertheless, alternative recycling techniques exist. The main limitation of classical mechanical recycling is the capacity of thermoplastic material to be melted and reused, since the polymeric structure is damaged after processing, with the polymeric length and properties of the material being negatively affected. Thus, the resulting properties and performances of the recycled materials are affected, limiting their application to low-value products and processes. In order to resolve these limitations in recycling, irradiation is proposed to regenerate the polymeric structure of the material and provide it with a comparative degree of crosslinking and properties when compared to the original. This approach avoids the decomposition of the original polyamide and its recombination into new plastic material, presenting the extra benefits of not introducing any contamination associated with chemical treatments and of avoiding the excess reagent removal step [10,11]. Moreover, the required time to recycle the materials is significantly reduced, lowering the carbon footprint impact, and the overall costs of the processes are reduced.

Currently, there are some effective energy radiation processes applied to plastics, such as electron beam [12] and gamma rays. The latter has been used as a technique for polymer upgrading over the last few decades with promising results [13]. The ionization induced by radiation is able to modify the polymer structure via two opposing mechanisms, namely chain scission and chain crosslinking, which imply direct changes in the structure and properties of the polymers [12,14]. While scission consists in breaking different covalent bonds across the polymeric structure, degrading the material by making the polymeric chains shorter, crosslinking leads to the recombination of different polymeric chains via generation of free radicals that in turn originate new bonds between different polymeric chains. These chains can be of the same or different polymeric structure. When they are different, the resulting material is a copolymer. The generation of these bonds is directly related to improvements in the mechanical and thermal properties of the material [15]. The present study is focused on PA6 monomaterial structure.

Most shortcomings associated with the thermomechanical processing of polyamides can be attributed to the chain scission mechanisms that take place during mechanical processing of the material with the consequent decline in molecular weight and properties, exactly as explained in the case of ionization. When these materials are treated with gamma rays, ionization induces crosslinking, revamping their properties and facilitating their recyclability.

During the irradiation process, free radicals are generated in the polymeric chains. These free radicals react among themselves, crosslinking the polymeric chains. Nevertheless, the atmosphere in which irradiation occurs (oxidizing or inert) plays a major role in the final process outcome, with various studies proving that scission prevails over crosslinking when irradiation takes place in air in the case of PA-6 [16], as oxygen takes part in secondary oxidation reactions. This is particularly relevant when using gamma rays since samples need to be irradiated for longer periods of time compared to other radiation sources to obtain the same dose. Thus, the probability of the formed radicals reacting with oxygen increases [17].

Gamma irradiation has been exploited by numerous industries as a way for obtaining upgraded materials for high-performance applications and also to increase compatibility between different polymer blends [18,19]. Although the results obtained are similar in nature to those attained through chemical crosslinking, in this case, they present the key advantage of not needing fillers to start the reactions, which avoids the use of toxic compounds [10,11]. Feasibility-wise, this technique has been regarded as a promising approach to polymer recycling when considering economical but also environmental points of view [20,21].

Within the present study, a systematical examination of the changes induced in the properties of raw and recycled PA-6 at different absorbed doses has been carried out. Both oxidizing and inert atmospheres have been used during radiation in order to understand the implications of employing each environment at the pre-industrial scale. Special attention has been paid to the changes in the rheological properties of the resulting materials, as these are their most important feature in processing and extrusion during polymeric product manufacturing. These changes in the rheological properties have been directly linked to the molecular weight and the effect of gamma rays on the molecular structure of the polyamide so the material changes could be linked from a chemical point of view to the practical use of the material in the industry.

This study is part of the polynSPIRE project (grant agreement No 820665) in which different strategies are being investigated towards the recycling of polyamides with chemical and mechanical technologies. All different technologies studied within the projects are applied in different sectors. In the case of the present study, the results will be demonstrated in the automotive sector, ascertaining the effect of different absorbed doses of gamma radiation in the recycling process of polyamide.

## 2. Materials and Methods

### 2.1. Materials

To evaluate whether gamma irradiation may be a feasible way to enhance the properties of degraded polyamide, a simulated residue—which from now on will be referred to as “recycled”—was obtained by extruding PA virgin material 7 times consecutively in a Coperion ZSK 26 twin screw extruder (Coperion, Stuttgart, Germany). Virgin PA-6 pellets were supplied by BADA (Bada Hispanaplast, Huesca, Spain), and the same extrusion parameters were kept for all extrusion processes; the temperature was set to 210 °C (entrance) to 245 °C (die), while the motor speed was settled at 150 rpm. The cooling down process was carried out in water at 25 °C, and the pick-up speed was 17 rpm. The material was dried after each extrusion at 85 °C for 5 h in order to eliminate the water attached to the surface of the PA before the next extrusion process. Samples were kept from each cycle so properties could be assessed as well as viscosity measurements used to understand the degradation and break of the polymeric chains, so it would be possible to resemble the conditions of a worn product. Materials were stored trying to replicate the conditions commonly employed in the plastic transformation industry.

A set of samples consisting of recycled and virgin or “raw” material were irradiated in air as well as an inert atmosphere (Argon), absorbing doses of 0, 100, 350 and 500 kGy. In the experiments, a Cobalt-60 gamma irradiation source was used. The equipment used for the irradiation of the samples at all different doses was a J.E.N 1970 irradiator (Aragogamma, Barcelona, Spain).

### 2.2. Characterization Methods

Different analytical techniques were employed to characterize the impacts on both the chemical and rheological properties of the polymer. 

The rheology of the samples was assessed using a INSTRON Ceast SR20 rheometer (Instron, Norwood, MA, USA). Since the viscosity of the material does not depend only on the shear rate but also on the temperature, it was necessary to perform the measurements at a temperature representative of the processing stages of the polymer. Accordingly, tests were carried out at 245 °C, a key temperature to observe the processability of the material (allowing extrusion and injection molding). The barrel has a diameter of 1.5 cm and a length of 35 cm. The capillary length is 20 mm with a diameter of 1 mm. The speed of the test was kept the same for all the tests performed (0.044-0.089-0.178-0.355-0.555-1.389-2.778-5.55-8.33 mm/s).

Molecular weight distributions of the samples were obtained via gel permeation chromatography (GPC) in a Waters 1515 instrument equipped with a Waters 996 PDA detector (Waters Corporation, Milford, CT, USA) and a Phenomenex Phenogel (Phenomenex, Torrance, CA, USA) 5 µm linear column (7.8 × 300 mm). The solvent used was hexafluoroisopropanol with a flow rate of 1 mL/min at 25 °C.

Thermogravimetric analyses were preformed using a NETZSCH TG 209F1 Libra (Netzsch, Selb, Germany) in a temperature range from 27 °C to 800 °C. The temperature increment rate was set at 10 °C/min.

A NETZSCH DSC 214 Polyma DSC was used to perform differential scanning calorimetry (DSC) in an inert atmosphere (N_2_). The temperature was raised from 25 °C to 300 °C and then cooled back down to 25 °C. For the third segment of the analysis, the temperature was increased again to 300 °C. The heat rate was kept to 10 °C/min in all cases. The glass transition temperature (Tg) was selected as the midpoint temperature value of the specific heat transition [22]. The degree of crystallinity was calculated by dividing the melting enthalpy of the second heating cycle by that corresponding to perfect PA-6 crystals (188 J/g) [23].

Electron paramagnetic resonance (EPR) was performed with a Bruker ELEXSYS 580 spectrometer (Bruker, Billerica, MA, USA), with a microwave frequency of 9.85 GHz, continuously and at ambient temperature. Samples were prepared as fibers with an approximate diameter of 2 mm. Experimental conditions were selected to avoid saturation and overmodulation: 6 mW of microwave power with a modulation amplitude of 0.15 mT and a modulation frequency of 100 Hz.

Fourier-Transform Infrared Spectroscopy (FTIR) was used to characterize the IR spectrum of the samples using a Vertex 70 (Bruker, Billerica, MA, USA). Pellets were applied a pressure of 1200 kg/cm^2^ over the course of 1 min to be crushed and analyzed. Study of the FTIR spectra focused on identifying the bands in Table 1 [24]. The wavelength range analyzed was from 400 to 4000 cm^−1^ (Mid-wavelength infrared), and the acquisition mode was attenuated total reflectance (ATR).

### 2.3. Data Analysis

Python was used to ascertain the dependence of the different variables on the irradiation dose absorbed. Scripts were developed to facilitate imports of the different results files (oftentimes found in different formats), homogenizing and pre-processing data. To estimate the degree to which crosslinking and scission occur after an irradiation event, several correlations have been proposed over the years, Charlesby–Pinner being the most widely adopted [25]. Both scission and crosslinking affect the molecular weight distribution of the polymer, and their yields may also be obtained through correlating the molecular weight distribution to the absorbed dose. This effect was analyzed using Equations (1) and (2), where $M_{n}$ are number average molecular weights, $M_{w}$ are weight averages, and G(S) and G(X) are the number of scission and crosslinking events per 100 eV, respectively [26]. The subindexes of the molecular weights express the absorbed dose D that the polymer has been exposed to. The radiolytic yields were obtained through identification of the slopes of the inverse of the molecular weights and solving the resulting linear system, ensuring dimensional coherence to express G(S) and G(X) in terms of events per 100 eV.

(1) $$\frac{M_{n,0}}{M_{n,D}}=1+\left[G\left(S\right)-G\left(X\right)\right]\frac{D}{100\;N_{A}}$$

(2) $$\frac{M_{w,0}}{M_{w,D}}=1+\left[G\left(S\right)/2-2\;G\left(X\right)\right]\frac{D}{100\;N_{A}}$$

Regarding viscosity, an analysis of the full results was undertaken to explore the influence of radiation on the rheological properties of the samples, aiming to produce a set of values able to describe the rheological behavior of the materials over the total range of shear rates provided by the rheometer, exploiting the data obtained in full.

To this end, the apparent viscosity (shear rate stress applied to a fluid divided by the shear rate) was plotted against the shear rate for virgin and recycled materials under varying absorbed doses, and accurate fittings for the data were provided following the power law (Ostwald–de Waele relationship) [27] as shown in equation 3, where η is the effective viscosity, $\dot{\gamma }$ is the shear rate, K is the flow consistency index, and n is the flow behavior index. Both the flow behavior (n) and the flow consistency (K) indexes were obtained making use of the optimization module of python’s SciPy library. This procedure was employed twice, as rheological results were available for material irradiated in air and in argon.

(3) $$\eta =K\;{\dot{\gamma }}^{n-1}$$

Dependence of the parameters K and n on the absorbed dose was also studied, providing mathematical expressions that allowed the calculation of both parameters from the absorbed dose of the experimental material. Through the use of this equation, it is possible to obtain the relationship between apparent viscosity and shear rate for any radiation level.

## 3. Results 

### 3.1. Rheological Characterization Results

**Figure 1:**
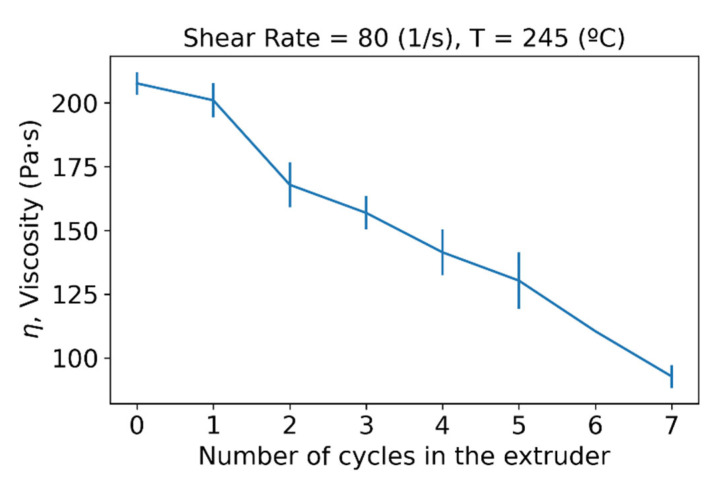
Viscosity variation with increasing number of cycles in the extruder.

The rheological properties of the samples were monitored after each extrusion process. Apparent viscosity measurements from the different extrusion cycles reveal that polymer suffers from degradation due to processing. Figure 1 shows the decreasing trend of viscosity with the increasing number of extrusions, reaching half its initial value once it had been processed seven times. Viscosity reductions can be explained through shorter molecular chains being formed through chain scission during the processing of the material [28,29,30].

**Figure 2:**
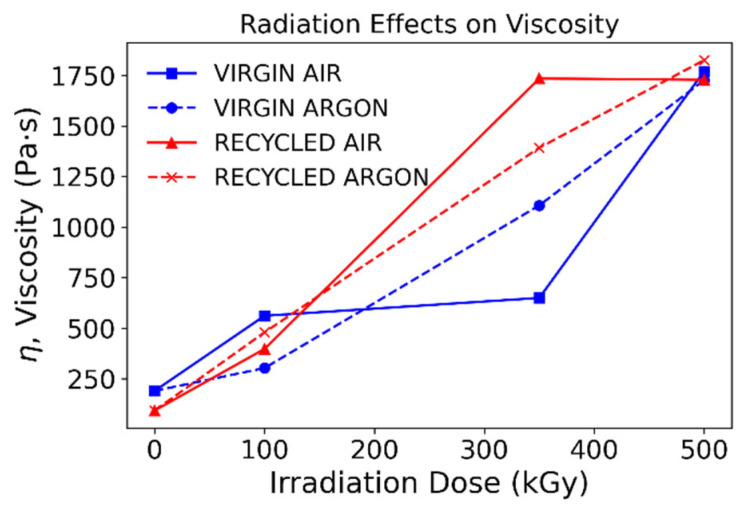
Radiation effects on viscosity.

As raw material and recycled material (extruded seven times) are irradiated, the viscosity values of the materials show a clear dependence on the absorbed doses of gamma irradiation. Apparent viscosities of samples irradiated show significant differences from the nonirradiated material, especially at higher doses, while variation at low irradiation levels is almost negligible (Figure 2). This increment in viscosity can be explained through branching in the lower doses and crosslinking at the higher. It can be observed that samples irradiated in argon show the lowest variability between recycled and virgin species, and this phenomenon is most likely linked to the randomness of the irradiation process when performed in air, as secondary oxidation reactions take place [17]. The recycled sample has a lower starting viscosity value, and the tendency is reverted at higher absorbed doses. The lower initial viscosity is not surprising since, as mentioned previously, mechanical degradation is likely to alter the initial structure of the recycled polymer by reducing the length of the chains. However, its value at higher absorbed doses could potentially be linked to crystalline memory behavior remaining at the work temperature and thus stronger associations between chains still existing. It is also possible that the initial impaired structure of the recycled polymer due to mechanical degradation provides nucleation sites for radical recombination and chain growth at doses where mixed effects are important. Values converge at 500 kGy where the absorbed dose is high enough for crosslinking to occur in both recycled and virgin polymers. However, viscosity values are increased one order of magnitude at high absorbed doses with respect to their initial state, radically limiting the materials’ processability with similar parameters to those set for the raw material.

**Figure 3:**
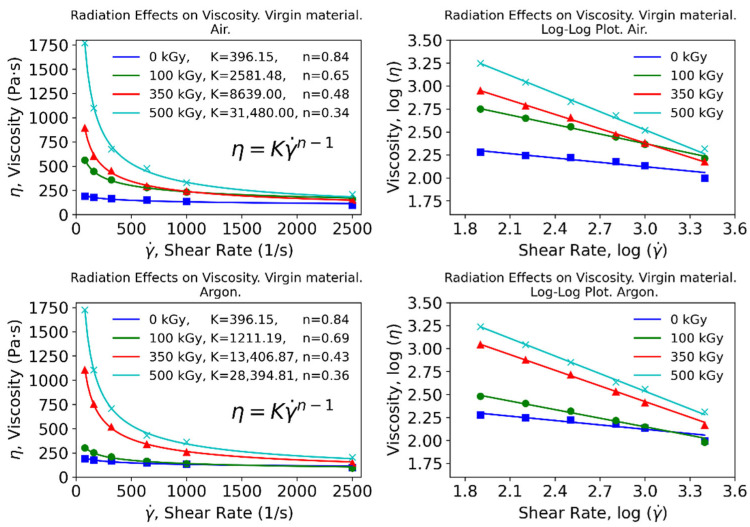
Apparent viscosity for virgin material at different absorbed doses. Top: irradiation in air. Bottom: inert atmosphere.

**Figure 4:**
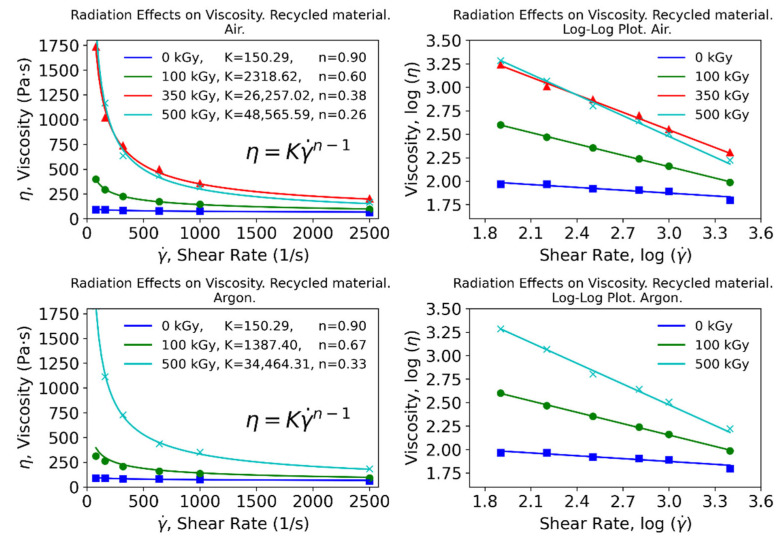
Apparent viscosity for recycled material at different absorbed doses. Top: irradiation in air. Bottom: inert atmosphere.

All data provided by the rheological tests were used in full to fit the apparent viscosity to the shear rate for samples subjected to different absorbed doses and under different conditions (air or inert atmosphere). As shown in Figure 3 and Figure 4, the use of python allowed calculation of the fitting parameters for a user-defined equation, which in this case was set to the power–law relationship, generating fittings that closely resembled the original data. For each case contemplated, additional log–log plots were produced where the same fitting is shown, resulting in close-to-straight lines for most cases.

**Figure 5:**
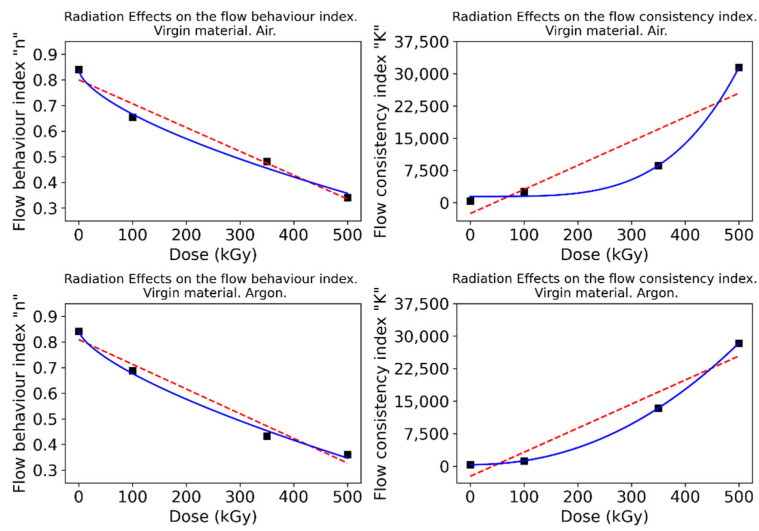
Dependence of the flow behavior and flow consistency indexes to the absorbed dose. Virgin material.

**Figure 6:**
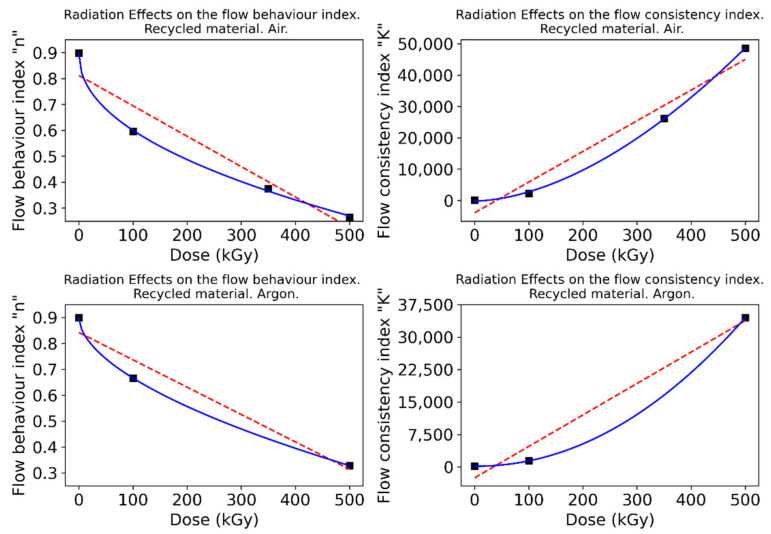
Dependence of the flow behavior and flow consistency indexes to the absorbed dose. Recycled material.

The resulting fittings show that the flow consistency index (K) is increased with the absorbed dose, while the flow behavior index (n) exhibits the opposite trend. Different fittings were implemented to gain insights from this relationship, including a linear regression and an exponential function to which a constant term is added for flexibility. Results for the virgin material are shown in Figure 5, while those corresponding to the recycled material are presented in Figure 6.

The exponential model fits the data to a high extent, resulting in high coefficients of determination that point to a well-defined trend for these parameters. Therefore, these correlations should serve as preliminary guidelines to estimate appropriate conditions in polymer processing itself, as well as to understand the underlying chemical phenomena.

The flow behavior index n has been traditionally regarded as an indicator of the nature of the fluid: when lower than 1 (Newtonian), it is considered a shear-thinning (pseudoplastic) fluid [31]. In view of the results, the fluid will show a more shear-thinning behavior with growing absorbed doses; however, this effect will produce greater changes in the nature of the fluid for moderate doses.

On the other hand, the flow consistency index K is related to the viscosity of the fluid. This parameter has been shown to increase exponentially with the absorbed dose. In summary, with increasing absorbed doses, more shear-thinning and viscous polymers are produced. At low doses, a significant decrease in n can be achieved at relatively little cost for K. Conversely, at high doses, a sharp increase in K can be obtained at low cost for n. Ultimately, the optimal combination would yield a viscosity value that is low enough to facilitate processing. At the same time, it must be emphasized that irradiation levels that yield viscosity values of both recycled and raw materials that show the lowest disparity would be optimal since, from a processing standpoint, it would be impossible to discern between the two. Achieving the latter is the goal pursued to validate the wide-scale deployment of this technique when reprocessing used polyamides.

### 3.2. Chemical Characterization Results

**Figure 7:**
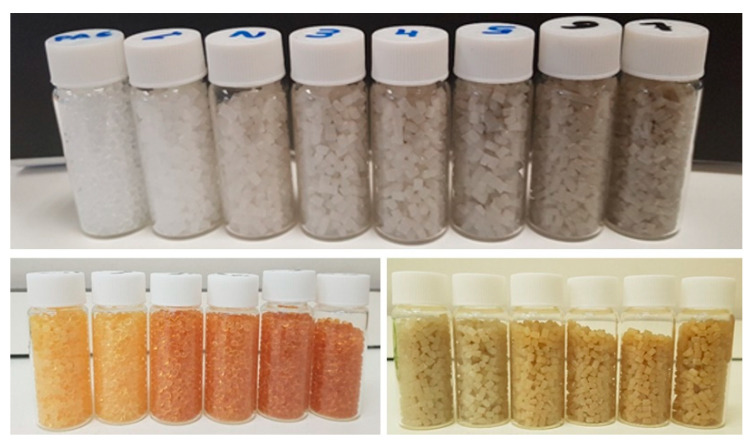
Top picture: virgin polyamide extruded up to seven times. Bottom left: irradiated virgin PA-6. Bottom right: Irradiated recycled PA-6.

The first observation that can be made regarding the chemistry of the samples is that the material became gradually darker as a result of mechanical and thermal stresses in the case of the simulated residue. A change in color also took place after irradiation of both virgin and recycled materials, as shown in Figure 7. In this case, the material became increasingly orange for growing absorbed doses. 

**Figure 8:**
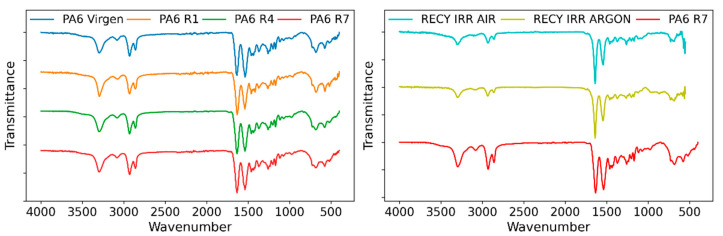
FTIR spectra. Left: virgin sample and samples extruded 1 (R1), 4 (R4) and 7 (R7) times. Right: recycled samples without irradiation and irradiated at 100 kGy in both air and argon.

This changes in the visible spectrum are also usually perceptible in the FTIR spectra. In the case of PA, it is important to analyze the peak intensity around 1710 and 1760 cm^−1^. To evaluate whether the fabrication process introduced significant thermal and mechanical degradation, the spectra for samples extruded one (R1), four (R4) and seven (R7) (recycled material) times were compared and contrasted, as shown in Figure 8.

Through this comparison, it was possible to carry out a qualitative analysis in which no major differences could be observed between samples; all reference peaks are present without appreciable discrepancies, agreeing with the current literature [32]. The analysis allows distinguishing the carbonyl group reference band as a mild slope in each sample, which confirms the existence of other compounds with a carbonyl functionality. 

Results were also compared to recycled samples irradiated at 100 kGy both in inert atmosphere and air, for which the same trends pointed out previously are observed (Figure 8). The smooth peak between 750 and 1000 cm^−1^ is greatly flattened in the irradiated specimens, while the peak at 1540 cm^−1^ is reduced and the peaks at 2500 cm^−1^ are also reduced. There seems to be no significant difference between those samples irradiated in air and argon. The carbonyl peak is still visible for all samples, and changes in color once again confirm oxidation phenomena taking place [33,34,35]. The growing color intensity with increasing absorbed dose is indicative of greater formation of chromophores [36].

**Figure 9:**
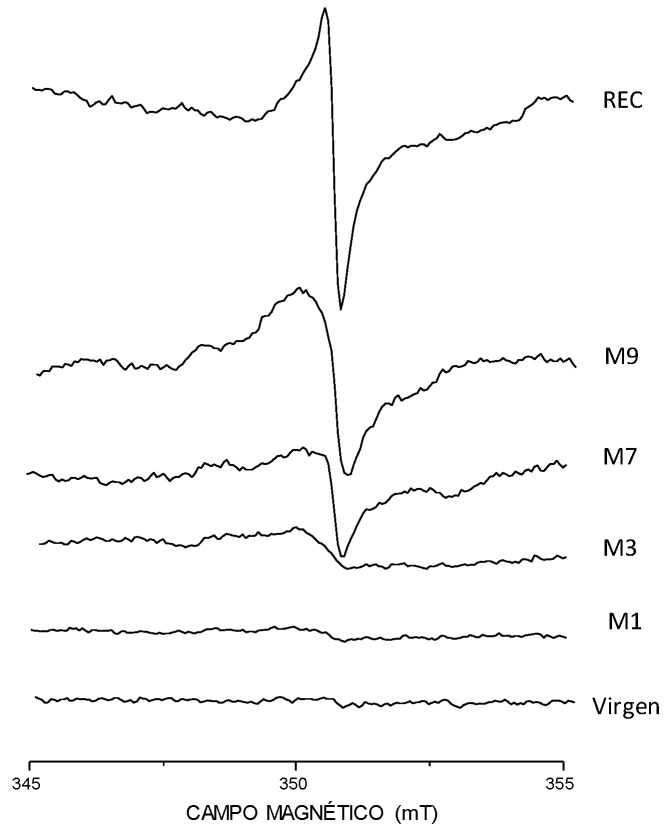
EPR spectra obtained for samples without and with irradiation at different absorbed doses. Scan in magnetic field: 345–355 mT.

EPR proved a viable technique to detect the presence of mechanically originated radicals, since although signals are all located in the same region, that corresponding to the non-irradiated recycled sample is broader and its shape somewhat different (Figure 9), allowing to verify through a different method that mechanical degradation indeed exists in re-processed samples. The samples analyzed in Figure 9 are specified in Table 2.

Location of the signal for the remaining samples is coherent with reported G-values in the literature (g = 2.00–2.20) [37] for the alpha amino methylene radical, and from a pure qualitative perspective, it may be possible to ascertain that the signal becomes more intense with increasing absorbed dose, as expected. The signals’ intensity in the recycled samples seems to be higher than in their raw counterparts for the same absorbed dose, with the intensity of the formed radicals in recycled samples being greater than for the virgin material [38].

**Figure 10:**
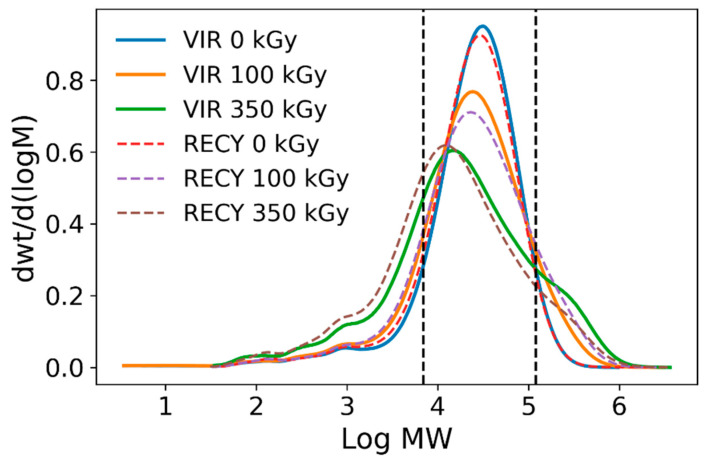
Plots of the molecular weight distributions obtained from GPC.

Despite the evident oxidative phenomena taking place, the goal of irradiating the different samples was to obtain chemical crosslinks that could bring the properties of the downgraded materials closer to those of the original PA-6. The first step in the characterization was therefore to evaluate whether crosslink formation was effective, and the absorbed doses required for this purpose. Figure 10 illustrates the results obtained from the GPC analyses in terms of molecular weight distributions.

**Figure 11:**
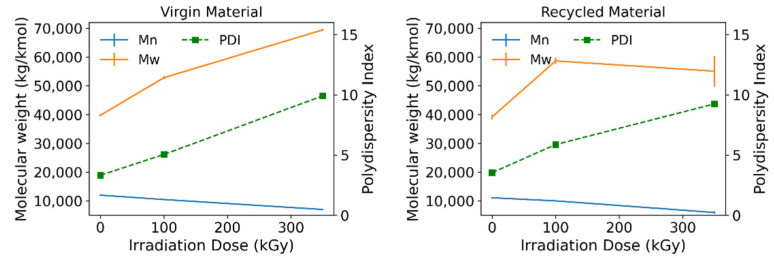
Weight and number average molecular weights for virgin (raw) and recycled material.

The molecular weight distributions as obtained by GPC for both the raw and recycled materials irradiated at different doses in air are shown in Figure 10. Values in Table 3 have been calculated making use of python’s scipy.integrate library to illustrate the proportion of the mass in the samples that fall within each of the marked MW intervals. Crosslinking and scission are even more apparent through these calculations, since it can be seen that upon irradiation, mid-sized molecules decrease in number, with scission being the predominant mechanism at 100 kGy. At 350 kGy, scission events are more significant in the recycled material, presenting a slight decrease in the crosslinking yield, whereas the amounts of large molecules keep rising steadily in the virgin polymer. Figure 11 summarizes the results on weight and number average molecular weights as well as the polydispersity indices obtained for both sets of samples. Results confirm simultaneous crosslinking and scission, given that the distribution broadens as the absorbed dose is increased. The shift of the distribution mode towards lower molecular weights indicates a predominance of scission over crosslinking over the entire irradiation range. However, the appearance of shoulder peaks at both sides of the maximum, especially at 350 kGy, does reveal that more chains have been subject to both effects at this absorbed dose. Several published sources do report that the gel formation dose is around 300 kGy [16,23]; therefore, the initial molecular weight increase and corresponding reduction in Mn can be attributed to chain branching as opposed to crosslinking. Chain growth seems to reach a plateau after 100 kGy for the recycled polyamide (note error bars at 350 kGy), which may have to do with a higher crystallinity degree, reducing chain mobility, and probability of crosslink formation in the amorphous regions. This seems particularly true for this case where the initial molecular weights distribution between raw and recycled polyamide is practically identical. Although it is a known fact that scission occurs while processing the recycled polyamide [30], variations in its initial molecular weight distribution are not significant enough to appreciate such a phenomenon in this case.

**Figure 12:**
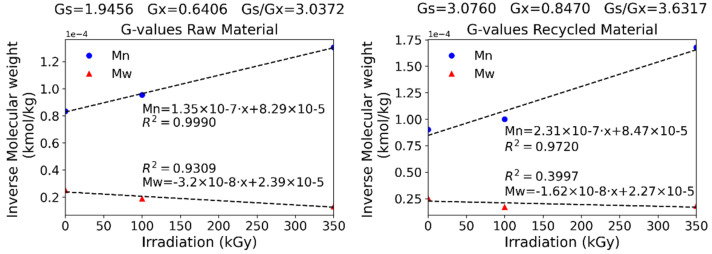
Radiolytic yields for virgin and recycled materials. G-values in (100 eV)^−1^.

Equations (1) and (2) were used to theoretically predict the scission and crosslinking effects of the irradiation process. The calculated G-values for both materials indeed verify a predominance of scission over crosslinking over the analyzed range, with scission being greater for the recycled material. Small variations in the initial distribution of the recycled polymer may have to do with its fabrication, since through extrusion melting, the polymer is subject to various thermal and mechanical stresses that may indeed reduce its stability and become oxidized at an earlier stage. As can be seen from Figure 11, the data fitting to a straight line are acceptable for the Mn correlation, whereas values deviate from a linear fitting when evaluating Mw. At the same time, it is clear from Figure 12 that the absorbed dose is being scanned over a range where different phenomena take place: at low absorbed doses, scission predominates, while at higher doses, crosslinking becomes more relevant. Adjusting single G-values for the whole range is therefore not entirely representative of the mechanisms occurring at each absorbed dose. Given that the Mn fitting is good enough for both groups of samples and so is the PD index (Table 4), Mw can be inferred for samples irradiated in this range. Results should only be taken as a guideline and used with caution, especially when dealing with recycled PA-6.

**Figure 13:**
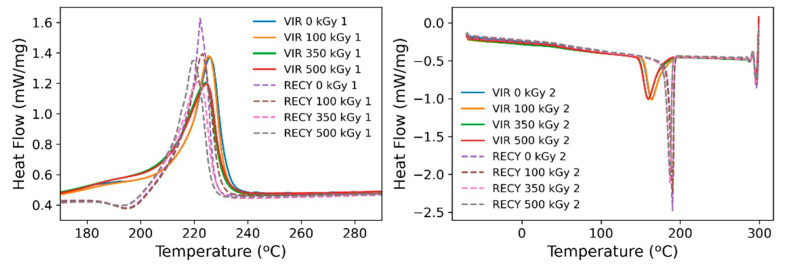
DSC Scans. Left: Melting peak for the first heating scan for both irradiated materials. Right: Crystallization peaks for both irradiated materials.

To corroborate the above findings, the DSC scans shown in Figure 13 were thoroughly analyzed. Changes in *Tg* are insignificant across the analyzed range, with changes in heat capacity showing the same trend. Invariance of *Tg* can be attributed to simultaneous crosslinking and scission since scission induces a reduction in *Tg*, while the opposite occurs for prevailing crosslinking. Tm shows a 2 °C drop for both cases when the absorbed dose reaches 350 kGy. The melting enthalpy is greater for recycled samples, which is indicative of a greater crystal portion as evidenced by their crystallinity. Typically, these changes are explained through crosslinking and branching taking place in the amorphous regions and boundaries of crystallites [23].

**Figure 14:**
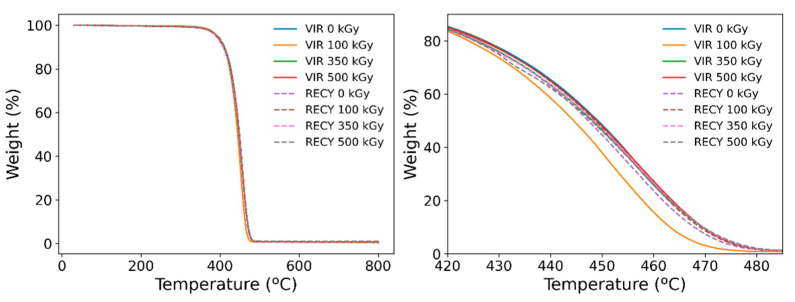
TGA scans for all samples. Left: whole analysis temperature range. Right: closeup of the intermediate range.

Crystallization temperatures are higher in the case of recycled polyamide, and so is the heat released during this process; the sharp exotherm peak observed for recycled samples illustrates the speed and temperature range of the process. At the same time, the crystallization temperature seems to decrease in both cases with increasing absorbed doses due to the delay in crystal formation produced by crosslinks [23]. It is clear that the shift in temperature observed between the recycled and virgin product reveals the presence of nucleation sites present in the recycled material, which favor rapid crystal formation. TGA scans (Figure 14) also reveal a higher thermal stability of the recycled products supporting this behavior. At the same time, recycled samples have been continuously melted and recrystallized at high temperatures for their fabrication, which may have introduced defects in the crystal portions that act as nucleation sites. Although the temperature has been raised over a range wide enough that should potentially melt most of the material, it is possible that continued thermal treatment during fabrication could have introduced memory effects in the material, and thus, crystallization tends to occur much faster. The latter effect has been reported elsewhere [32].

The first heating scan shows a shoulder to the left of the main melting peak for raw polyamide, which disappears during the second run, while the exact opposite occurs for recycled polyamide. These shoulders appear as a result of the co-existence of different crystal forms in the polymer or changes in the crystallite thickness and distribution [32]. While it seems that the controlled melting and subsequent cooling of the samples homogenize the crystallite structure in the case of virgin polyamides, the sharp crystallization peak over a narrower temperature range in the case of recycled material may induce the formation of different crystal forms.

In order to evaluate the effect of the processing conditions and irradiation on thermal stability, TGA analysis was performed under the conditions described in the experimental section. The main characteristics for the scans are summarized in Table 5. The results support previous hypotheses on polymeric chain behavior. The material presenting faster weight loss is the raw (virgin) sample irradiated at 100 kGy. It is known that scission events coexist with crosslinking under these irradiation conditions, and thus, chain rupture and polymer degradation result in chemical species that tend to decompose easily [17]. The main difference with its recycled counterpart is that crystallinity in this sample was about 20% higher; thus, bonds in the latter configuration are stronger. Scission during processing can also lead to gel formation, creating stronger chemical interactions in the material [39].

The concurrence of scission and crosslinking has been assessed by the GPC, leading to broader molecular weight distributions, reinforcing the conclusions of the TGA. Taking into account that the viscosity vales were raised with increasing absorbed doses and contrasting these with the GPC distributions, a possible explanation for the revamping of the rheological values under concurrent scission and crosslinking is that while some polymeric chains are shortened through scission, the ones undergoing crosslinking have a dominant effect when regarding rheological properties. This would explain why, even when scission takes place, it is still possible that crosslinking is the dominant mechanism affecting the rheological properties.

## 4. Conclusions

In this research, we demonstrated the capacity and potential of gamma irradiation and its effect on recycled materials at a simulated industrial scale. While thermoplastic materials are processed several times, the length of their polymeric structure is reduced, impacting their mechanical properties and resulting in a reduction in viscosity that dramatically changes the processing parameters and final performance.

Nevertheless, this research was not only focused on the analysis of recycled PA-6 processability enhancement after subjection to multiple stresses, but also on assessing the chemical behavior of the irradiated PA-6 that resembles that of the original polymer. These two objectives have been addressed independently but while looking for a common solution and strategy.

The use of gamma radiation at a 100 kGy dose has been demonstrated to be able to recover the viscosity loss in the material through the generation of free radicals, which result in crosslinking. Since this viscosity is similar to that of raw polymer, changing the processing conditions of the extrusion can be avoided, and a fraction of recycled polymer cam be processed at the same time as new polymer. However, future work should investigate the effect of gamma radiation at lower absorbed doses in the range from 0 to 100 kGy. 

Further, it has been shown that the effect of oxygen does not impact the chemical and rheological properties of the material to an extent that could render the use of Argon a cost-effective way of upgrading the recycled material. After characterization of both virgin and recycled samples, it can be concluded that gamma irradiation in air is a suitable way to restore the original characteristics of polyamide 6 from the point of view of processing, although further research is needed in order to make this process economically viable. One way of achieving this would be focusing on the effects at lower absorbed doses, especially in the range from 0 to 100 kGy, since to achieve a 100 kGy dose, about a day of irradiation is needed.

## Figures and Tables

**Table 1:** FTIR most significant reference bands for this study.

Compound	Wavelength (cm^−1^)
Ketones	1715
Aldehydes	1725
Aliphatic carboxylic acids	1750
Esters	1735
CH_2_ scissor bond	1458–1468

**Table 2:** EPR spectra obtained for samples without and with irradiation at different absorbed doses. Scan in magnetic field: 345–355 mT.

Spectrum Reference	Sample
Virgen	Virgin PA—no irradiation
M1	Virgin PA—100 kGy irradiation in air
M3	Virgin PA—350 kGy irradiation in air
M7	Recycled PA—100 kGy irradiation in air
M9	Recycled PA— 350 kGy irradiation in air
Rec	Recycled PA—no irradiation

**Table 3:** Mass fractions for the sections split by the vertical dashed lines illustrated in Figure 10. Expressed as percentages.

	Log MW < 3.84	3.84 < Log MW < 5.08	Log MW > 5.08
VIRGIN 0 kGy	12.24	83.18	4.58
VIRGIN 100 kGy	16.11	74.13	9.76
VIRGIN 350 kGy	26.52	58.93	14.55
RECYCLED 0 kGy	13.43	82.10	4.47
RECYCLED 100 kGy	16.64	70.80	12.56
RECYCLED 350 kGy	32.12	57.19	10.69

**Table 4:** Summary of fitting results of GPC data.

	Virgin PA-6	Recycled PA-6
G-value crosslinking (100 eV)^−1^	0.6406	0.8470
G-value scission (100 eV)^−1^	1.9456	3.0760
Mn correlation	1.35 × 10^−7^ D + 8.29 × 10^−5^	2.31 × 10^−7^ D + 8.47 × 10^−5^
Mn determination coefficient	0.9990	0.9720
PD correlation	1.90 × 10^−2^ D + 3.2475	1.58 × 10^−2^ D + 3.8498
PD determination coefficient	0.9993	0.9813
Mw correlation	−3.20 × 10^−8^ D + 2.39 × 10^−5^	−1.62 × 10^−8^ D + 2.27 × 10^−5^
Mw determination coefficient	0.9309	0.3997

**Table 5:** Main characteristics of the TGA analysis.

	T 10	T Onset	T Max	Residue %
VIRGIN 0 kGy	410.89	373.39	479.67	0.677
VIRGIN 100 kGy	409.30	370.98	473.20	0.645
VIRGIN 350 kGy	408.42	370.60	479.64	0.783
VIRGIN 500 kGy	409.84	371.00	479.24	0.830
RECYCLED 0 kGy	409.10	371.40	477.19	1.131
RECYCLED 100 kGy	411.62	372.58	477.63	1.114
RECYCLED 350 kGy	407.36	370.58	478.85	0.896
RECYCLED 500 kGy	410.55	371.40	479.69	0.981

## Data Availability

The data presented in this study are available on request from the corresponding author. The data are not publicly available due to containing information that could compromise the privacy of research participants.
